# Supplementary material for: From sequence to enzyme mechanism using multi-label machine learning
Source: BMC Bioinformatics. 2014 May 19;15:150. doi: 10.1186/1471-2105-15-150 (PMC4229970; doi:10.1186/1471-2105-15-150)
Supplement: Additional file 2 — Java code of ml2db. Additional file ml2db_code.tar.gz contains the Java source code to run the multi-label machine learning experiments and save the results to database. The code’s Javadoc is included. [file 1471-2105-15-150-S2.zip › additional file 2/ml2db/ml2db/doc/main/ml2db/mulan/learn/crosseval/EvaluationResultsTable.html]

EvaluationResultsTable


JavaScript is disabled on your browser.


- Overview
- Package
- Class
- Use
- Tree
- Deprecated
- Index
- Help

- Prev Class
- Next Class

- Frames
- No Frames

- All Classes

- Summary:
- Nested |
- Field |
- Constr |
- Method

- Detail:
- Field |
- Constr |
- Method


main.ml2db.mulan.learn.crosseval

## Class EvaluationResultsTable

- java.lang.Object
- - uk.ac.standrews.utils.main.database.Table
  - - main.ml2db.mulan.learn.crosseval.EvaluationResultsTable

- ---

    

  ```
  public class EvaluationResultsTable
  extends uk.ac.standrews.utils.main.database.Table
  ```

  Gives the names and table field names for multi-label specific evaluation
  measures (such as micro-accuracy, macro-accuracy, hamming loss etc.)

  Version:
  :   8 Jun 2010

  Author:
  :   Luna De Ferrari ldf(at)st-andrews.ac.uk

- - ### Field Summary

    Fields

    | Modifier and Type | Field and Description |
    | --- | --- |
    | `static java.lang.String` | `ALGORITHM` the name for the algorithm table column |
    | `static java.lang.String` | `END_TIMESTAMP` time at end of computation |
    | `static java.lang.String` | `EXPERIMENTS_TABLE_NAME` the name for the table to store the evaluation results |
    | `static java.lang.String` | `RUN` the evaluation run number (eg: between 0 and 9 for a 10 fold cross evaluation) |
    | `static java.lang.String` | `TEST_ARFF_ID` the table column to store the arff file id |
    | `static java.lang.String` | `TESTSET_LABELS` |
    | `static java.lang.String` | `TRAIN_ARFF_ID` the table column to store the arff file id |

    - ### Fields inherited from class uk.ac.standrews.utils.main.database.Table

      `m_writer`
  - ### Constructor Summary

    Constructors

    Constructor and Description

    `EvaluationResultsTable(uk.ac.standrews.utils.main.database.DbManager dbManager)`
  - ### Method Summary

    Methods

    | Modifier and Type | Method and Description |
    | --- | --- |
    | `java.util.TreeMap<java.lang.String,java.lang.String>` | `addEvaluationResults(java.util.TreeMap<java.lang.String,java.lang.String> data, mulan.evaluation.Evaluation results)` adds the evaluation results to the data map (table column 2 value map) |
    | `static java.util.Vector<uk.ac.standrews.utils.main.database.TableColumn>` | `getColumnDefinitions()` Add timestamps and identifiers to the cross evaluation results |
    | `static java.util.Hashtable<java.lang.String,java.lang.String>` | `getMeasureFieldNames()` A hashtable containing for each evaluation measure the corresponding table field name. |
    | `static java.util.Hashtable<java.lang.String,uk.ac.standrews.utils.main.database.TableColumn>` | `getMeasureFields()` A hashtable containing for each measure field name the corresponding table field object (name + sql data type). |
    | `static java.lang.String` | `getMeasureTableField(java.lang.String evaluationMeasureName)` For each evaluation measure, gets the corresponding table field The field name is the evaluation measure name, all lower-case, with spaces and dashes substituted with underscores. |
    | `static void` | `main(java.lang.String[] args)` |
    | `int` | `saveRow(java.lang.Object[] data)` Save the evaluation results to table. |

    - ### Methods inherited from class uk.ac.standrews.utils.main.database.Table

      `getColumnNames, getColumns, getDbManager, getTableCreator, getTableName, getTableReader, getTableRow, getTableWriter, hasKey, initialise, initialise, name, saveRow`
    - ### Methods inherited from class java.lang.Object

      `equals, getClass, hashCode, notify, notifyAll, toString, wait, wait, wait`

- - ### Field Detail


    - #### ALGORITHM

      ```
      public static final java.lang.String ALGORITHM
      ```

      the name for the algorithm table column

      See Also:
      :   Constant Field Values


    - #### TRAIN\_ARFF\_ID

      ```
      public static final java.lang.String TRAIN_ARFF_ID
      ```

      the table column to store the arff file id

      See Also:
      :   Constant Field Values


    - #### TEST\_ARFF\_ID

      ```
      public static final java.lang.String TEST_ARFF_ID
      ```

      the table column to store the arff file id

      See Also:
      :   Constant Field Values


    - #### END\_TIMESTAMP

      ```
      public static final java.lang.String END_TIMESTAMP
      ```

      time at end of computation

      See Also:
      :   Constant Field Values


    - #### EXPERIMENTS\_TABLE\_NAME

      ```
      public static final java.lang.String EXPERIMENTS_TABLE_NAME
      ```

      the name for the table to store the evaluation results

      See Also:
      :   Constant Field Values


    - #### RUN

      ```
      public static final java.lang.String RUN
      ```

      the evaluation run number (eg: between 0 and 9 for a 10 fold cross
      evaluation)

      See Also:
      :   Constant Field Values


    - #### TESTSET\_LABELS

      ```
      public static final java.lang.String TESTSET_LABELS
      ```

      See Also:
      :   Constant Field Values
  - ### Constructor Detail


    - #### EvaluationResultsTable

      ```
      public EvaluationResultsTable(uk.ac.standrews.utils.main.database.DbManager dbManager)
      ```
  - ### Method Detail


    - #### addEvaluationResults

      ```
      public java.util.TreeMap<java.lang.String,java.lang.String> addEvaluationResults(java.util.TreeMap<java.lang.String,java.lang.String> data,
                                                                              mulan.evaluation.Evaluation results)
      ```

      adds the evaluation results to the data map (table column 2 value map)


    - #### saveRow

      ```
      public int saveRow(java.lang.Object[] data)
      ```

      Save the evaluation results to table.

      Parameters:
      :   `data` -

      Returns:
      :   the identifier of the table row


    - #### getColumnDefinitions

      ```
      public static java.util.Vector<uk.ac.standrews.utils.main.database.TableColumn> getColumnDefinitions()
      ```

      Add timestamps and identifiers to the cross evaluation results

      Parameters:
      :   `run` -
      :   `arffId` -
      :   `startTime` -
      :   `endTime` -
      :   `learner` -
      :   `evaluationResults` -
      :   `tableManager` -

      Returns:
      :   a full evaluation record
          public int saveRow(String run, String startTime, String endTime,
          MulanLearner learner, Evaluation evaluationMetrics, String
          trainArffId, String testArffId) { TreeMap row =
          new TreeMap();
          // generate results row row.put(DbUtils.START\_TIMESTAMP,
          startTime);
          row.put(EvaluationResultsTable.TRAIN\_ARFF\_ID, trainArffId);
          row.put(EvaluationResultsTable.TEST\_ARFF\_ID, testArffId);
          row.put(EvaluationResultsTable.ALGORITHM,
          learner.getLearnerFullName());
          row.put(EvaluationResultsTable.RUN, run);
          // end timestamp row.put(DbUtils.END\_TIMESTAMP, endTime);
          // add evaluation results TreeMap fullRow =
          this.addEvaluationResults(row, evaluationMetrics);
          return super.saveRow(fullRow);
          }


    - #### getMeasureFieldNames

      ```
      public static java.util.Hashtable<java.lang.String,java.lang.String> getMeasureFieldNames()
      ```

      A hashtable containing for each evaluation measure the corresponding
      table field name.

      Returns:
      :   a 'measure name-to-field name' hash table


    - #### getMeasureFields

      ```
      public static java.util.Hashtable<java.lang.String,uk.ac.standrews.utils.main.database.TableColumn> getMeasureFields()
      ```

      A hashtable containing for each measure field name the corresponding
      table field object (name + sql data type).

      Returns:
      :   a 'field name-to-field' hash table


    - #### getMeasureTableField

      ```
      public static java.lang.String getMeasureTableField(java.lang.String evaluationMeasureName)
      ```

      For each evaluation measure, gets the corresponding table field The field
      name is the evaluation measure name, all lower-case, with spaces and
      dashes substituted with underscores.

      Returns:
      :   a column name taken from a Mulan
          `Measure` name


    - #### main

      ```
      public static void main(java.lang.String[] args)
      ```


- Overview
- Package
- Class
- Use
- Tree
- Deprecated
- Index
- Help

- Prev Class
- Next Class

- Frames
- No Frames

- All Classes

- Summary:
- Nested |
- Field |
- Constr |
- Method

- Detail:
- Field |
- Constr |
- Method
